# Evolving Practice Patterns in the Use of Prophylactic Cranial Irradiation for Extensive-Stage Small Cell Lung Cancer

**DOI:** 10.1001/jamanetworkopen.2019.9135

**Published:** 2019-08-14

**Authors:** Olsi Gjyshi, Ethan B. Ludmir, Todd A. Pezzi, David Boyce-Fappiano, Amy E. Dursteler, Timur Mitin, Steven H. Lin

**Affiliations:** 1 Department of Radiation Oncology, The University of Texas MD Anderson Cancer Center, Houston; 2 Department of Radiation Medicine, Oregon Health & Science University, Portland

## Abstract

This survey study compares use of prophylactic cranial irradiation in patients with extensive-stage small cell lung cancer before and after the publication of a phase 3 trial that demonstrated no overall survival benefit for this therapy.

## Introduction

The recent phase 3 trial by Takahashi et al^1^ showed no overall survival benefit with prophylactic cranial irradiation (PCI) over active magnetic resonance imaging (MRI) surveillance among patients with extensive-stage small cell lung cancer (ES-SCLC), questioning the previously established benefit of PCI for this patient group.^2^ Consequently, in 2018, the National Comprehensive Cancer Network^3^ established equipoise between MRI surveillance and PCI in ES-SCLC. These developments have fueled debate over the role of PCI in this setting, and current practice patterns remain unclear.^4^ We conducted a nationwide survey study of radiation oncologists (ROs) to assess changes in the use of PCI for patients with ES-SCLC following publication of the trial by Takahashi et al.^1^

## Methods

We invited all American Society for Radiation Oncology–registered, US-based ROs to answer a survey addressing their use of PCI for patients with ES-SCLC before and after publication of the study by Takahashi et al.^1^ The survey, which was designed and analyzed in accordance with American Association for Public Opinion Research (AAPOR) reporting guideline, was administered anonymously, with responder identifiers automatically replaced with random identifiers, and stored in a password-protected database.^5^ Written informed consent was obtained at the beginning of the survey. The participants were invited to participate in an anonymous, voluntary survey intended for academic purposes. The study was not submitted for institutional review board approval according to the guidelines established by the Federal Policy for the Protection of Human Subjects, 45 CFR part 46. Pearson χ^2^ testing assessed for differences in proportions and binary logistic regression was used to estimate the factors associated with PCI use. All statistical tests performed were 2-sided, and a *P* value less than .05 was considered statistically significant. Analyses were conducted using SPSS statistical software version 24.0 (IBM).

## Results

In total, 3851 ROs were invited to participate in the survey, with 487 participants (12.6%) completing the survey (Figure). Baseline characteristics were generally well distributed (Table). Of 487 respondents, 454 (93%) were aware of the study by Takahashi and colleagues.^1^ While 72% of them reported that they routinely offered PCI to ES-SCLC patients prior to the publication of the trial by Takahashi et al,^1^ only 44% continue to do so currently (difference, 28%; 95% CI, 25%-31%; *P* < .001) (Figure). There was no difference in current practice patterns between academic and private practice ROs (43% vs 45%; difference, 2%; 95% CI, −7% to 11%; *P* = .71) (Figure). Regression analysis showed no difference in likelihood of offering PCI based on practice setting, location, or size; volume of patients treated for lung cancer; or years in practice (Table). Of the respondents not aware of the study by Takahashi et al,^1^ 85% continued to offer PCI, a higher percentage than those who were aware of the study (odds ratio, 0.11; 95% CI, 0.04-0.32; *P* < .001) (Figure). Regarding future research, 47% of all respondents reported willingness to enroll both patients with limited-stage SCLC and ES-SCLC in a randomized clinical trial comparing MRI surveillance with PCI; 15% would enroll only patients with limited-stage SCLC, and 20% would enroll only patients with ES-SCLC.

**Figure.**
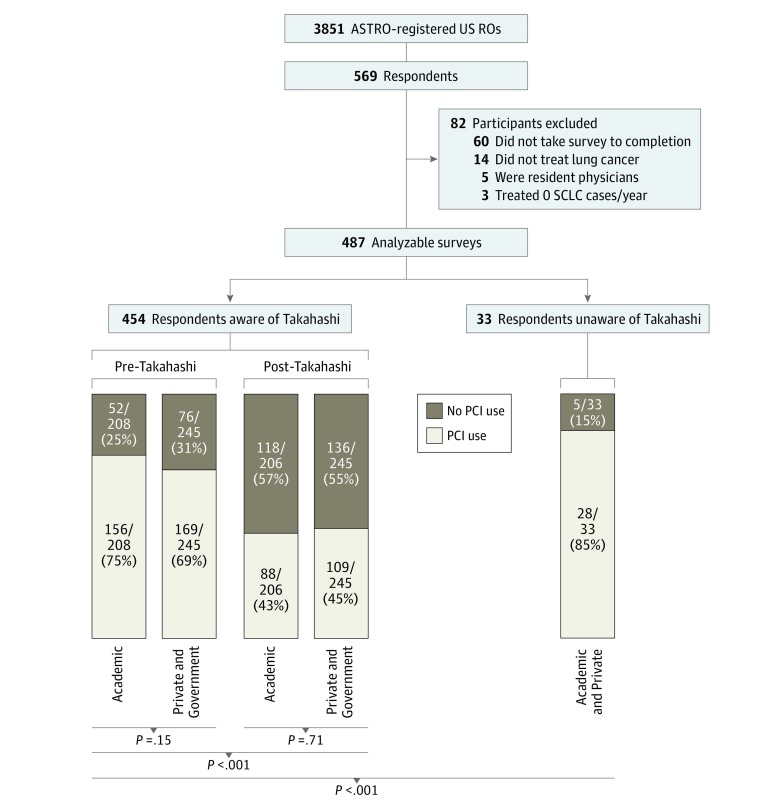
Schematic of Survey and Responses All American Society for Radiation Oncology (ASTRO)–registered US-based radiation oncologists (ROs) were invited via email to participate in an anonymous survey. To avoid recall bias, all respondents were initially asked if they currently routinely offer prophylactic cranial irradiation (PCI) to patients with extensive-stage small cell lung cancer (ES-SCLC). Then they were asked if they were aware of the study by Takahashi et al.^1^ The 454 respondents who were aware of the study were then asked if they offered PCI prior to the study (lower left section of Figure); this question was omitted for the 33 respondents not aware of the study using a branching logic system, and only current PCI use was reported for these respondents (lower right section of Figure).

**Table.** Respondent Demographic Characteristics and Pattern of PCI Use

Demographic Characteristic	No. (%)			Multivariate Analysis^a^	
	Respondents	Currently Offering PCI			
		No	Yes	OR (95% CI)	*P* Value
Practice environment					
Academic	216 (44)	119 (56)	95 (44)	1 [Reference]	
Private practice or government	270 (56)	140 (52)	130 (48)	1 (0.64-1.56)	.99
Aware of trial by Takahashi et al^1^					
No	33 (7)	5 (15)	28 (85)	1 [Reference]	
Yes	454 (93)	255 (56)	197 (44)	0.11 (0.04-0.32)	<.001
Geographic location					
Northeast	100 (21)	53 (53)	47 (47)	1 [Reference]	
Midwest	122 (25)	67 (55)	54 (45)	0.81 (0.43-1.55)	.53
South	114 (24)	56 (50)	57 (50)	0.66 (0.35-1.23)	.19
West	69 (14)	44 (64)	25 (36)	0.94 (0.49-1.79)	.85
Other	80 (16)	40 (50)	40 (50)	0.62 (0.30-1.26)	.19
Practice size, No.					
2-5	188 (39)	91 (48)	97 (52)	1 [Reference]	
6-10	129 (27)	66 (52)	62 (48)	1.67 (0.85-3.29)	.14
11-20	99 (21)	60 (61)	38 (39)	1.53 (0.78-2.99)	.21
>20	65 (14)	39 (60)	26 (40)	0.97 (0.48-1.96)	.94
Lung cancer cases treated annually, No.					
1-25	189 (39)	100 (53)	88 (47)	1 [Reference]	
26-50	169 (35)	93 (55)	76 (45)	1.44 (0.72-2.9)	.30
>50	129 (26)	67 (52)	62 (48)	1.05 (0.59-1.88)	.87
Years in practice					
1-5	142 (29)	90 (63)	52 (37)	1 [Reference]	
6-10	88 (26)	40 (46)	47 (54)	0.75 (0.37-1.54)	.44
11-20	108 (17)	53 (49)	55 (51)	1.61 (0.76-3.39)	.21
21-30	98 (16)	50 (52)	47 (48)	1.18 (0.57-2.42)	.66
>30	51 (10)	27 (53)	24 (47)	1.08 (0.52-2.28)	.83
Sex					
Male	378 (88)	203 (54)	173 (46)	1 [Reference]	
Female	104 (22)	54 (52)	50 (48)	0.80 (0.50-1.31)	.39

Abbreviations: OR, odds ratio; PCI, prophylactic cranial irradiation.

^a^ A multivariate binary logistic analysis was used to determine association between the variables.

## Discussion

Prior to the study by Takahashi and colleagues,^1^ previous data demonstrated a 98% rate of offering PCI for patients with ES-SCLC.^6^ The present survey demonstrates a reduction in the use of PCI for ES-SCLC; knowledge of the study by Takahashi et al^1^ appears to be the driving force for this shift, as lack of awareness of the study was associated with significantly higher rates of continued PCI use. This evolution of practice was observed similarly in both academic and nonacademic settings, as well as all other tested demographic groups, emphasizing the widespread changes associated with the trial.

Notably, most respondents (82%) were willing to enroll patients with limited-stage SCLC and/or ES-SCLC in a trial comparing MRI surveillance with PCI. These results highlight the continued lack of consensus for PCI in SCLC and support ongoing investigations such as the proposed SWOG-1827 trial attempting to investigate PCI vs MRI surveillance in all patients with SCLC.

Participation bias is a potential limitation of our study, as participants aware of the study by Takahashi et al^1^ may have been more inclined to complete the survey, resulting in possible overestimation of the true awareness of the trial and underestimation of current PCI use in the United States.

This survey found that US physician respondents reported a reduced rate of PCI use in ES-SCLC following publication of the study by Takahashi and colleagues,^1^ and most expressed interest in further research on the topic.
